# Immune Response to SARS-CoV-2 Vaccination in Cancer Patients: A Prospective Study

**DOI:** 10.7759/cureus.37014

**Published:** 2023-04-01

**Authors:** Cecília Caramujo, Inês Gomes, Teresa Fraga, Judy Paulo, Sofia Broco, Nuno Cunha, Pedro Madeira, Teresa Carvalho, Margarida Teixeira, Gabriela Sousa

**Affiliations:** 1 Medical Oncology, Instituto Português de Oncologia de Coimbra Francisco Gentil, E.P.E., Coimbra, PRT; 2 Pathology, Instituto Português de Oncologia de Coimbra Francisco Gentil, E.P.E., Coimbra, PRT

**Keywords:** covid-19, immune-response, chemotherapy, cancer, sars-cov-2

## Abstract

Introduction

Cancer patients on active treatment are at increased risk of developing coronavirus disease 2019 (COVID-19), making effective immunization of the utmost importance. However, the effectiveness of vaccination in this population is still unclear. This study aims to evaluate the response against COVID-19 in a cohort of patients with active cancer under immunosuppressive therapy.

Methods

This was a prospective, cross-sectional, single-center study that included patients with cancer under immunosuppressive therapy vaccinated against COVID-19 between April and September 2021. Exclusion criteria were: previous known severe acute respiratory syndrome coronavirus 2 (SARS-CoV-2) infection, single-dose vaccine or incomplete vaccination scheme. Immunoglobulin G (IgG) anti-SARS-CoV-2 antibody levels were assessed using 35.2 binding antibody units (BAU)/mL as the positive cut-off. Assessments were performed 14-31 days after the first and second dose and three months after the second dose.

Results

A total of 103 patients were included. The median age was 60 years. Most patients were being treated for gastrointestinal cancer (n=38, 36.9%), breast cancer (n=33, 32%) or head and neck cancer (n=18, 17.5%). At evaluation, 72 patients (69.9%) were being treated with palliative intent. The majority were being treated with chemotherapy (CT) alone (57.3%). At the first assessment, levels of circulating SARS-CoV-2 IgG consistent with seroconversion were present in 49 patients (47.6%). At the time of the second assessment, 91% (n=100) achieved seroconversion. Three months after the second dose, 83% (n=70) maintained levels of circulating SARS-CoV-2 IgG consistent with seroconversion. In this study, no SARS-CoV-2 infection was reported in the study population.

Conclusions

Our findings suggest that this group of patients had a satisfactory COVID-19 immunization response. Although promising, this study should be replicated on a wider scale in order to validate these findings.

## Introduction

In December 2019, a new coronavirus (severe acute respiratory syndrome coronavirus 2 [SARS-CoV-2]) emerged and with it its disease, coronavirus disease 2019 (COVID-19), a severe acute respiratory syndrome with high morbidity and mortality, and was declared a global pandemic by the World Health Organization in March of 2020 [1].

It is known that certain comorbidities, such as active cancer, can increase susceptibility to COVID-19. In cancer this increase in susceptibility is due to the state of immunosuppression that it induces and that is either due to the treatments used, as a side effect, or to specific characteristics of the tumor itself such as location of the primary tumor or the extent of the disease [2,3]. As such, patients with cancer are not only at a greater risk for severe forms of COVID-19 but are also associated with higher mortality rates [3]. In a recent meta-analysis that included 52 studies, with a total of 18,650 cancer patients with COVID-19, the mortality rate was 25.6% [4].

In Portugal, vaccination against COVID-19 is being made with the administration of the COVID-19 vaccine from Pfizer (two doses, 28 days apart), Moderna (two doses, 28 days apart), Janssen (one dose), and AstraZeneca (two doses, 12 weeks apart). With the exception of the AstraZeneca vaccine, which is made up of a genetically modified viral vector with no replicative capacity, and therefore with no contraindication in immunosuppressed patients, the other two approved vaccines consist of a non-replicating nucleic acid (messenger ribonucleic acid [mRNA]) [5-7]. Efficacies in preventing the symptomatic disease of COVID-19 in the clinical trials that led to the approval of the COMIRNATY, Moderna and AstraZeneca vaccines ranged from 95%, 94.1%, and 90%, respectively [8,9].

There is limited information on the effectiveness and safety of licensed SARS-CoV-2 vaccines in patients with cancer since most clinical trial protocols did not include them [3]. Overall, the reported seroconversion rate in patients with solid tumors is about 90% [10-16]. Moreover, the efficacy of the vaccines in patients with solid tumors is less clear given the small number of patients and the inclusion of patients with hematologic malignancies in a few studies which limited the evaluation of the effects of chemotherapy, immunotherapy, and biologic treatments in this subgroup of patients with solid tumors [1,17,18].

By extrapolating data from other vaccines, such as the influenza virus vaccine, it may be safe to assume that the safety of the SARS-CoV-2 vaccine in cancer patients will be similar to that of the rest of the population [3]. Even so, similarly to what happens with the influenza vaccine, the seroconversion and protection conferred seem to be lower in patients undergoing chemotherapy [8]. On the other hand, immunological checkpoint inhibitors, due to their mechanism of action, seem to be associated with a greater immune response to vaccines, as already demonstrated with the use of anti-influenza vaccines [19].

The purpose of this work is to evaluate the serological response (through the quantification of anti-SARS-CoV-2 immunoglobulin G [IgG] antibodies in the blood) in patients undergoing systemic therapy for a solid tumor, in order to evaluate and monitor the response to immunization, namely its effectiveness, as well as adverse effects.

Preliminary results were previously presented at the 18th Congresso Nacional de Oncologia on November 20th, 2021.

## Materials and methods

Study design

This was a prospective, single-center study, approved by the Institutional Ethics Committee (Comissão de Ética do Instituto Português de Oncologia de Coimbra Francisco Gentil; approval 23/2021) and conducted in accordance with the principles of the Declaration of Helsinki and the International Conference on Harmonization Good Clinical Practice guidelines. Between April and September 2021, patients with an active solid malignant tumor under antineoplastic therapy (chemotherapy, monoclonal antibody-based therapy, cyclin-dependent kinase inhibitors, immunotherapy, tyrosine kinase inhibitor) at the time of vaccination against COVID-19 were tested for circulating antibody anti-SARS-CoV-2. Serologic response was defined as antibody levels (IgG) >35.2 binding antibody units (BAU)/mL. The blood samples were collected 14-31 days after the first and second doses and at the three, six, and nine months mark after the second dose. Collected sera were analyzed for SARS-CoV-2 IgG using the anti-SARS-CoV-2 QuantiVac assay (Euroimmun, Lübeck, Germany).

Study population

Patients who were at least 18 years old, had histological confirmation of an active solid cancer, and were under antineoplastic therapy at the time of vaccination against COVID-19 were recruited. Patients with known previous SARS-CoV-2 infection, patients that were vaccinated with a single dose vaccine or those who did not complete the vaccination scheme were excluded.

Study assessments and outcomes measures

Medical records of the included patients were reviewed. The following information was recorded: demographic data, Eastern Cooperative Oncology Group Performance Status (ECOG-PS), location of primary tumor, staging of the disease at the time and type and therapeutic intent of antineoplastic therapy prescribed. Circulating anti-SARS-CoV-2 antibodies were measured 14-31 days after the first and second doses and three months after.

Statistical analysis

Data analysis was completed using Statistical Package for the Social Sciences (SPSS) Statistics version 25 (IBM Corp., Armonk, NY, USA). Descriptive statistics for all continuous variables were reported as mean (standard deviation) or median (inter-quartile range), depending on the normality of distribution. Categorical variables were reported as counts and percentages.

## Results

A total of 103 patients were included. Table 1 shows the demographic and baseline characteristics of the patients. The median age was 60 years (min 38, max 84). The majority of patients were female (n=55; 53.4%) and had an ECOG performance status of <2 in 94.1% (n=98). Most patients were being treated for gastrointestinal cancer (n=38, 36.9%), breast cancer (n=33, 32.0%) or head and neck cancer (n=18, 17.5%). The remaining 14 patients were being treated either for lung cancer (n=6, 5.8%), gynecological cancer (n=4, 3.9%), urological cancer (n=2, 1.9%), pancreatic cancer (n=1, 1.0%) or skin cancer (n=1, 1.0%). At evaluation, 72 patients (69.9%) were being treated with palliative intent. Sixteen patients were receiving neoadjuvant treatment (15.5%) and 15 were on adjuvant treatment.

**Table 1:** Demographic and baseline clinical characteristics of the patients Abbreviations: CDK4/6, cyclin-dependent kinases 4 and 6; ECOG-PS, Eastern Cooperative Oncology Group Performance Status; TKI, Tyrosine-kinase inhibitor.

Variable	Subgroup		n	(%)
Age	≤65 years		66	(64.1)
	>65 years		37	(35.9)
Sex	Male		48	(46.6)
	Female		55	(53.4)
ECOG-PS	0		57	(55.3)
	1		41	(39.8)
	2		5	(4.9)
Primary cancer location	Gastrointestinal		39	(37.9)
	Breast		33	(32.0)
	Head and Neck		18	(17.5)
	Lung		6	(5.8)
	Gynecological		4	(3.9)
	Urologic		2	(1.9)
	Skin		1	(1.0)
Status	Metastatic disease	72		(69.9)
	Local disease		31	(30.1)
Treatment	Chemotherapy		59	(57.3)
	Chemotherapy + Antibody		17	(16.5)
	CDK4/6 inhibitor		12	(11.7)
	Immunotherapy		7	(6.8)
	Tyrosine-kinase inhibitor		4	(3.9)
	Antibody		3	(2.9)
	Chemotherapy + TKI		1	(1.0)
Treatment goal	Palliative		71	(68.9)
	Neoadjuvant		16	(15.5)
	Adjuvant		16	(15.5)
Type of Covid-19 vaccine	Messenger RNA-based		94	(91.3)
	Viral vector		9	(8.7)

The majority were being treated with chemotherapy (CT) alone (57.3%) and 17 (16.5%) with CT together with monoclonal antibody-based therapy (vascular endothelial growth factor [VEGF] and epidermal growth factor receptor [EGFR] inhibitors). As for the remaining 26.3% of patients they were on cyclin-dependent kinase (CDK) inhibitor (n=12, 11.7%), immunotherapy (n=7, 6.8%), tyrosine kinase inhibitor (TKI) (n=4, 3.9%), antibody monotherapy (n=3, 2.9%) and CT with TKI (n=1, 1.0%).

Concerning the vaccine itself, 94 patients (91.3%) received a messenger RNA-based COVID-19 vaccine and only nine patients (8.7%) received a viral vector COVID-19 vaccine. Seventy-eight (75.7%) of these vaccines were produced by Pfizer BioNTech, 16 (15.5%) by Moderna and nine (8.7%) by AstraZeneca.

SARS-CoV-2 IgG levels prior to vaccination were assessed in 26 patients (25.2%), and two patients had circulating SARS-CoV-2 IgG. At the first assessment, levels of circulating SARS-CoV-2 IgG consistent with seroconversion were present in 49 patients (47.6%). The median levels were 21.81 BAU/mL (<1 - 29799). At the time of the second assessment, three patients were lost to follow-up (two deaths and one to hospice care). As for the remaining population (n=100), 91.0% achieved seroconversion. Median levels were 923.85 BAU/mL (<1 - 35742.30).

In the evaluation of seroconversions after the second dose of the vaccine by the therapeutic group (n=91), it was found that the group of patients receiving chemotherapy had a percentage of seroconversion of 87.3% compared with 95.5% in the group of patients treated with other antineoplastic therapies (antibodies, immunotherapy, and tyrosine kinase inhibitors). The mean value of IgG antibodies was lower in the group of patients treated with chemotherapy alone when compared with other therapies (2594.44 BAU/mL versus 2739 BAU/mL). 

**Figure 1:**
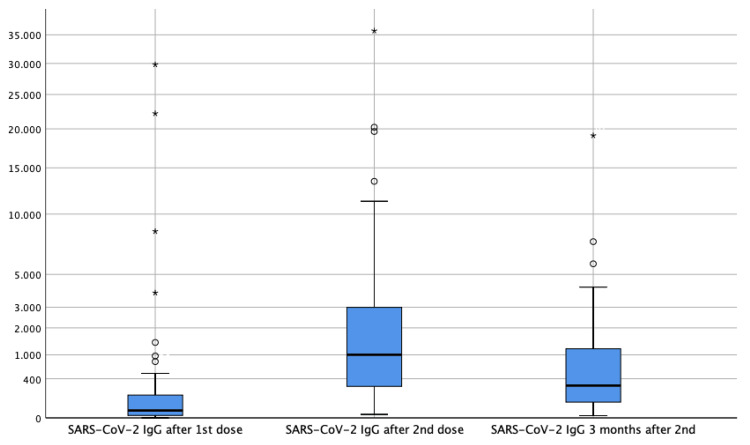
Boxplot showing the distribution of SARS-CoV-2 IgG values 14-30 days after the first dose, 14-30 days after the second dose and three months after the second dose of COVID-19 vaccine. IgG: Immunoglobulin G

Three months after the second dose 16 patients were lost to follow-up (four deaths, five to hospice care, and five dropouts). Of the remaining 84 patients, 83.0% (n=70) maintained levels of circulating SARS-CoV-2 IgG consistent with seroconversion (Figure 1). Median levels were 275.12 BAU/mL (<1 - 19089.84). Regarding the therapeutic groups, IgG antibody values remained with a lower mean value in patients treated with chemotherapy when compared to patients treated with other therapies (968.26 BAU/mL versus 1648.04 BAU/mL).

Only two patients were under corticosteroid therapy at the time of vaccination. The one patient receiving the highest dose of corticosteroid (more than 4mg of dexamethasone equivalent dose) did not achieve levels of circulating SARS-CoV-2 IgG consistent with seroconversion throughout the time of evaluation. The other patient under corticosteroid therapy (less than 4mg of dexamethasone equivalent dose) achieved seroconversion in the first evaluation and maintained it throughout the remaining evaluations.

In this study, no SARS-CoV-2 infection was reported in the study population.

## Discussion

We currently have several COVID-19 vaccines authorized worldwide, namely two mRNA-based vaccines, BNT162b2 produced by Pfizer-BioNTech and mRNA-1273 by Moderna, and two vaccines produced using adenovirus vectors, AZD1222 by Oxford-AstraZeneca and Ad26.COV2.S by Janssen, which have demonstrated efficacy in producing immunogenicity against SARS-CoV-2, with acceptable safety profiles [18,20]. However, cancer patients were known to be grossly under-represented in the approval studies, and the large-scale studies demonstrating the efficacy of the second dose of vaccine in preventing COVID-19 either did not provide data for patients on anti-neoplastic therapy or excluded individuals on immunosuppressive therapy [18,21,22].

Existing data on the immunogenicity of the COVID-19 vaccine in cancer patients are preliminary and limited to post-vaccine antibody levels (to the viral spike protein) [16]. It has been documented that the vast majority of patients with solid tumors receiving chemotherapy generate immune responses after the two doses of vaccine, and it is also perceptible that, despite seroconversion, levels may be lower than in the control population without cancer [16,23].

In the cohort of patients in this study, 49 (47.6%) of 103 patients receiving anticancer treatment had anti-IgG levels compatible with seroconversion 14-31 days after the first dose and 91% of patients were seropositive for anti-IgG at 14-31 days after the second dose of one of the three vaccines (from Pfizer BioNTech, Moderna and AstraZeneca). These results suggest that these patients can produce IgG in response to the SARS-CoV-2 vaccine, but that a second dose is essential, which is consistent with studies published in the literature, such as the Israeli study by Massarweth et al., in which only patients with solid cancer were included, with a positive serological response in 90% of the 102 patients who were on active anti-neoplastic therapy [16]. However, the IgG titers dosed after the second dose of vaccine were much lower than healthy controls reported in the literature (923.85 BAU/mL in the study population vs 3577-23628 BAU/mL) [16,24].

Regarding the maintenance of the immunological response to vaccination, when the IgG levels values were analyzed three months after the second dose of anti-SARS-CoV-2 vaccine, the number of patients with positive seroconversion decreased to 83% (70 out of 83 patients), with a significant drop in the mean value of IgG levels to 275.12 BAU/mL. When we compare these values with those of the general population (median values reported in the literature of 440 u/mL), we can see that the levels in our population of cancer patients who have received the vaccines while under anti-neoplastic therapy are much lower [25].

Overall, we could see that in the 91 patients evaluated after the second dose of the vaccine, the chemotherapy group had a lower percentage of seropositivity and lower mean IgG levels (87.3%; 2596.44 BAU/mL) than patients on other anti-neoplastic therapies (95.5%; 2739.0 BAU/mL) (antibodies, immunotherapy, and tyrosine kinase inhibitors). This pattern of poorer response in patients on chemotherapy is also seen when IgG levels are measured three months after the second dose, with a difference of minus 1.7x compared to patients on other treatments.

In this study, during the monitoring period, no SARS-CoV-2 infection was reported in the study population, with only one patient showing a clear elevation in the antibody titer dose at three months (1268,85 BAU/mL after the second dose to 3529,91 BAU/mL at three months) which could correlate with an asymptomatic SARS-CoV-2 infection.

This study showed significantly lower anti-S IgG SARS-CoV2 antibody levels in cancer patients on anti-neoplastic therapy when compared to the values of healthy individuals described in the existing literature. At present, the correlation between IgG levels and protection against COVID-19 is not well established, and data regarding antibody levels required to neutralize the virus are not yet available [16]. Recently, a cohort study demonstrated that patients with positive antibody test results were initially more likely to have positive nucleic acid amplification test (NAAT), consistent with prolonged RNA loss; but markedly less likely to have positive nucleic acid amplification test results over time, suggesting that seropositivity is associated with protection against infection [26,27].

One bias is that our sample was not evenly distributed per cancer type and treatment. Due to the small sample size, no statistically significant correlation was found between the various subgroups. However, it would be important to study this in a larger cohort of patients.

## Conclusions

This study serves as a sample of the encouraging results in immunological response to the vaccine against COVID-19, although these were slightly lower than in the general population represented in some studies.

As the correlation between antibody levels after vaccination and clinical protection is not yet established, future research is needed to determine the level and duration of protection conferred by vaccines in cancer patients. In any case, this study suggests that vaccination of cancer patients while on anticancer therapy is safe and should be prioritized. Although promising, in order to validate these findings, this study needs to be reproduced on a larger scale.
